# Memory-Like NK Cells: Remembering a Previous Activation by Cytokines and NK Cell Receptors

**DOI:** 10.3389/fimmu.2018.02796

**Published:** 2018-11-28

**Authors:** Jens H. W. Pahl, Adelheid Cerwenka, Jing Ni

**Affiliations:** ^1^Department for Immunobiochemistry, Universitätsmedizin Mannheim, Medizinische Fakultät Mannheim, Universität Heidelberg, Mannheim, Germany; ^2^Innate Immunity, German Cancer Consortium, German Cancer Research Center (DKFZ), Heidelberg, Germany

**Keywords:** memory, NK cell, IL-12 cytokines, IL-12/15/18, CD16, recall (memory), adoptive transfer, immunotherapy

## Abstract

Natural Killer (NK) cells are cytotoxic innate lymphoid cells serving at the front line against infection and cancer. In inflammatory microenvironments, multiple soluble and contact-dependent signals modulate NK cell responsiveness. Besides their innate cytotoxic and immunostimulatory activity, it has been uncovered in recent years that NK cells constitute a heterogeneous and versatile cell subset. Persistent memory-like NK populations that mount a robust recall response were reported during viral infection, contact hypersensitivity reactions, and after stimulation by pro-inflammatory cytokines or activating receptor pathways. In this review, we highlight recent findings on the generation, functionality, and clinical applicability of memory-like NK cells and describe common features in comparison to other recent concepts of memory NK cells. Understanding of these features will facilitate the conception and design of novel NK cell-based immunotherapies.

## Introduction

NK cells were discovered in the 1970s, when it was concluded that NK cells are able to naturally lyse certain tumor target cells without prior sensitization and mediate lysis of antibody-opsonized tumor cells (1). NK cells were characterized to respond to cells that have a loss (“missing-self”) or reduced levels in cognate self-MHC class I molecules and, thus, are unable to engage inhibitory NK cell receptors, contrary to the MHC class I–restricted recognition of foreign antigens by cytotoxic T cells (2, 3). Nowadays, it is well-established that the threshold for NK cell cytotoxicity is dictated by the surplus of activating over inhibiting signals from target cells and the microenvironment (4). A multitude of NK cell activating and inhibitory surface receptors regulates target cell elimination and the production of immunostimulatory cytokines like IFN-γ. Aside from their classical innate immune functionality, NK cells can promote adaptive immune responses or elicit regulatory functions under certain conditions (5). For instance, tissue-resident decidual NK cells can give rise to a specifically enriched NK cell subset upon repeated pregnancies, exerting enhanced IFN-γ and VEGF production, which might improve placentation (6, 7). The uncovering of immunological memory has added to the complexity of NK cell biology (8). Following MCMV infection, murine NK cells acquire traits of adaptive immunity such as expansion of virus/m157-specific NK cell subsets and long-lasting enhanced secondary responses including improved protection against MCMV compared to that of naïve NK cells (9). NK cells can exert antigen-specific memory against previously sensitized haptens or viruses in a T/B cell-independent manner (10). In essence, virus and hapten-specific memory NK cells in mice resemble antigen-specific immunological memory of T and B cells to some extent with phases of expansion and contraction but a more limited selection of antigen-specific recall responses (11). Similarly, human NKG2C^+^ NK cells enriched in patients with a history of HCMV infection, referred to as “adaptive” NK cells, may rely at least in part on the specific recognition of HLA-E–loaded HCMV peptides (12, 13). Besides NK cells, allergen- or IL-33-experienced group 2 innate lymphoid cells acquire antigen unspecific memory-like properties (14).

The cytokines IL-2 and IL-15 drive NK cell differentiation, proliferation, and activation, while IL-15 trans-presented by activated dendritic cells is critical for NK cell survival (15–17). IL-2 has long been known to prime the cytotoxic function of NK cells toward cancer cells (18). IL-2 produced by antigen-specific memory CD4 T cells has been implicated in NK cell activation during anti-viral recall responses (19–21). IL-12 and IL-18 secreted during viral infections by e.g., dendritic cells induce potent NK cell IFN-γ production and cytotoxicity, in particular in combination, and synergistically augment IL-2 and IL-15–induced NK cell activation (20, 22–26). The direct effects of cytokine-mediated NK cell activation may involve a reduced threshold of activating receptor signaling (27–31), increased expression of activating receptors (32, 33), a lower (but not absent) responsiveness to cognate inhibitory MHC class I ligands (34, 35), a ready-to-execute cytotoxic machinery by granule convergence (36), and changes in NK cell metabolism (31, 37).

**Figure 1:**
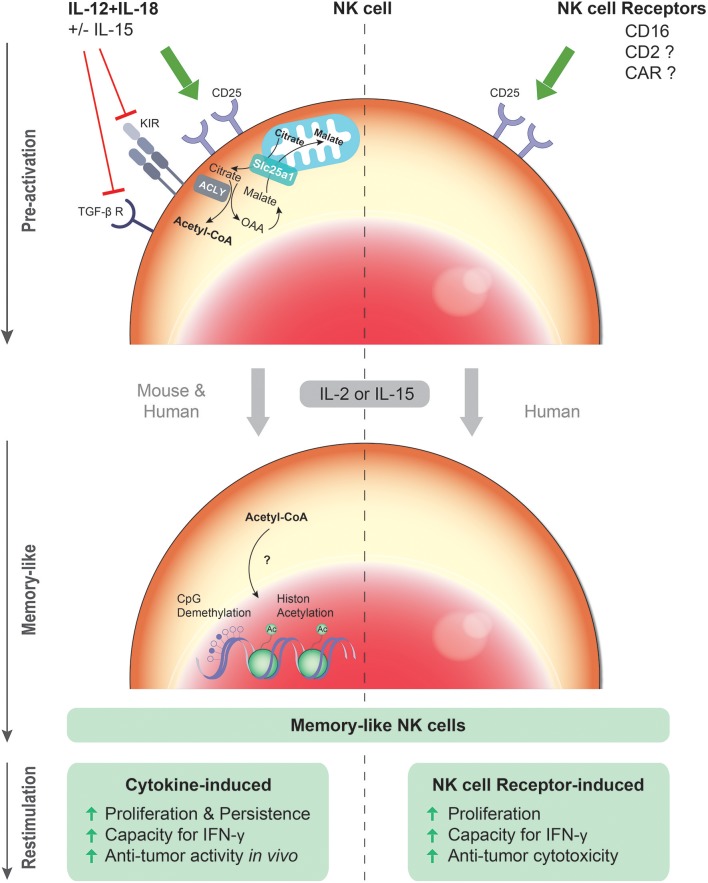
Cytokine- and NK cell receptor-induced memory-like NK cells. Upon primary exposure to the cytokine combination IL-12/18 plus IL-15, murine and human NK cells up-regulate the IL-2 receptor α chain (CD25), and undergo rapid proliferation and expansion in response to IL-2 or IL-15. Moreover, down-regulation of the TGF-β receptor and certain inhibitory KIRs by IL-12/15/18 might contribute to the superior effector function of the cytokine pre-activated NK cells. After restimulation with cytokines or tumor cells, these cytokine pre-activated NK cells have an enhanced capacity to produce IFN-γ and a more robust and sustained anti-tumor activity *in vivo*. Epigenetic modification such as CpG demethylation and histone acetylation (Ac) induced by the cytokines might be crucial for the persistent competence of enhanced gene transcription upon restimulation. Similarly, upon primary exposure to ITAM-associated NK cell activating receptors such as CD16, human NK cells undergo in response to IL-2 or IL-15 more rapid proliferation and expansion *in vitro*. After restimulation with cytokines or tumor cells, these CD16 pre-activated NK cells have an enhanced capacity to produce IFN-γ and a more robust cytotoxic activity. Hence, both cytokine-induced and CD16-induced memory-like functionalities are antigen-unspecific and share the property of “remembering” a previous activated state induced by cytokine or antibody exposure.

In addition to the direct effects of cytokines on NK cell activation, there is emerging evidence that pre-activation by IL-12 and IL-18 plus IL-15 can endow murine and human NK cells with long-lasting enhanced NK cell functionality even after discontinuation and in the absence of the initial stimulus (Figure 1). This so-called memory-like functionality is antigen-unspecific and characterized by an enhanced proliferative capacity, prolonged persistence *in vivo* for up to 3 months, and superior IFN-γ production and potent cytotoxic activity upon *ex vivo* restimulation (8, 38). The generation, mechanistic insight, physiological relevance and therapeutic potential of antigen-unspecific memory-like NK cells are the prime focus of this review.

## Properties of memory-like NK cells

### Cytokine-induced memory-like functionality

In 2009, Cooper et al. demonstrated that mouse NK cells pre-activated with the cytokine cocktail IL-12, IL-15, and IL-18 persisted at high numbers several weeks after transfer into RAG-1^−/−^ mice and produced higher levels of IFN-γ upon restimulation *ex vivo* compared to control NK cells (39). Later, our group and others showed that mouse and rat IL-12/15/18 pre-activated NK cells could mount a more robust and long-lived anti-tumor response after adoptive transfer (40, 41). This memory-like NK cell activity required extrinsic help from IL-2 producing CD4 T cells and was associated with intrinsic demethylation of the *IFNG* locus, facilitating IFN-γ transcription and production upon restimulation (42).

Analogous to murine NK cells, activation of human NK cells with IL-12/18 plus IL-15 for 16 h conferred memory-like functionality after *in vitro* re-culture in IL-15 or IL-2 for several days. IL-12/15/18 pre-activated NK cells produced more IFN-γ upon restimulation with cytokines, K562 cells or primary acute myeloid leukemia (AML) blasts in comparison to control NK cells, which had been pre-activated with an equivalent dose of IL-15 (40, 43) or with low-dose IL-15 (44). Importantly, 6 days after transfer into tumor-free T/B/NK cell-deficient NSG mice (supplemented daily with IL-2), IL-12/15/18 pre-activated NK cells were superior in IFN-γ production when restimulated *ex vivo* with K562 cells or cytokines (24, 42, 44). In xenograft mouse models, adoptively-transferred IL-12/15/18 pre-activated NK cells significantly ablated melanoma growth in the lung (42) and reduced systemic K562 tumor burden associated with improved survival (44).

NK cells pre-activated with IL-12/18 +/− IL-15 were more sensitive to low concentrations of IL-2 due to increased surface density of the high-affinity IL-2 receptor α chain (CD25) (Figure 1), resulting in more rapid proliferation and a higher NK cell recovery upon IL-2 culture (24, 40). Accordingly, in an immunocompetent tumor microenvironment, IL-12/15/18 pre-activated NK cells might be superior in competing for low amounts of IL-2 with CD25^+^ regulatory T cells, which restrain IL-2–dependent expansion of NK cells and T cells after adoptive cell transfer (45, 46). Of note, IL-2 was critical for the profound proliferation of IL-12/15/18 pre-activated NK cells, their anti-tumor activity and persistence in several organs such as blood, spleen, liver, and lung after adoptive transfer (42). IL-2 may be provided by host CD4 T cells activated by homeostatic proliferation in tumor-bearing non-lethally irradiated mice (40). Furthermore, the concerted activation of CD4 T cells and myeloid cells co-transferred within autologous PBMC could substitute IL-2 injections after adoptive transfer (42).

Directly after cytokine stimulation, IL-12/15/18 pre-activated NK cells mediated more potent cytotoxicity as compared to IL-15 activated NK cells (42, 47). Of note, this difference may be more pronounced against target cells displaying cognate self-MHC class I ligands, since IL-12/15/18 pre-activation for at least 48 h has been shown to reduce inhibitory KIR expression (35) (Figure 1). The difference compared to IL-15 pre-activated NK cells might merely reflect a prolonged state of potent activation. After *in vitro* re-culture, low-dose IL-15 pre-activated NK cells exhibited lower DNAM-1-dependent cytotoxicity against primary AML blasts than IL-12/15/18 pre-activated NK cells (44). In contrast, degranulation of NK cells pre-activated with IL-12/15/18 or an equivalent dose of IL-15 was comparable against NK cell-sensitive K562 cells (43), which are mainly recognized through the NK cell receptors NKG2D and NKp30 (48, 49). Thus, it remains to be resolved whether IL-12/15/18 pre-activated memory-like NK cells, i.e., when restimulated after adoptive transfer or after re-culture with IL-2 or IL-15 *in vitro*, indeed possess higher cytolytic activity, in particular against target cells that are poorly killed by IL-2 or IL-15 pre-activated or resting NK cells. Interestingly, IL-12, IL-15, and IL-18 are known to down-regulated the TGF-β receptor and its signaling pathway (Figure 1). Hence, resistance to TGF-β might add to the potency of IL-12/15/18-induced memory-like NK cells (50). Moreover, it has been reported that IL-12 and IL-18 injections reversed the anergic phenotype of NK cells in tumor-bearing mice (51). Whether this treatment would induce memory-like properties *in vivo* in endogenous NK cells is unknown. In spite of the up-regulation of several surface markers such as CD25, CD69, KLRG1 on a more mature CD11b^high^CD27^low^ NK cell subpopulation (40, 52) and down-regulation of the KIRs as well as the TGF-β receptor, an unequivocal biomarker profile is lacking to discriminate IL-12/15/18 pre-activated NK cells *in vivo*.

### NK cell receptor-mediated memory-like functionality

Cancer cells might confer contact-dependent priming signals through NK cell activating receptors that enhance NK cell activation. It has been suggested that NK cells (cultured without/with IL-2/15) displayed enhanced cytotoxicity after a previous co-culture with certain tumor cells, although the exact priming stimulus was not identified (53, 54). In this context, it has been shown that pre-activation of NK cells with tumor cells through CD2 and its ligand CD15 on tumor cells could enable subsequent lysis of otherwise poorly susceptible target cells (55). CD2 is a co-stimulatory receptor for ITAM-coupled NK cell receptors like NKp46 and CD16 (29, 56). Upon cross-linking CD2 can associate with CD3ζ signaling by forming a complex with CD16 at the immunological synapse (57), and CD2 can lead to STAT5 phosphorylation similar to IL-2 and IL-15 (58, 59). While the adoptive transfer of these tumor cell-primed NK cells has been tested in a phase 1 study in AML patients (60), it is unknown whether priming of NK cells by tumor cells can occur *in vivo* in an NK cell immunosuppressive microenvironment.

Recently, our group has revealed that FcγRIIIa/CD16 engagement by therapeutic (bispecific) antibodies can prime enhanced memory-like NK cell functionality in addition to direct activation known as classical antibody-dependent cellular cytotoxicity (ADCC). Following 5-day IL-2 re-culture, CD16 pre-activated NK cells exerted enhanced antibody-independent cytotoxicity and IFN-γ production upon restimulation with cytokines or tumor cells compared to IL-2 cultured NK cells (47) (Figure 1). Similar to cytokine-induced memory-like NK cells, CD16 pre-activated NK cells up-regulate CD25 expression (Figure 1) in particular in the presence of IL-12, resulting in increased sensitivity to low-dose IL-2 and more vigorous proliferation and expansion in response to IL-2 (47, 61, 62). Potential common mechanisms of cytokines and CD16-engaging antibodies in inducing memory-like NK cells require further investigation. Altogether, the concept of CD16-induced memory-like functionality of NK cells needs to be investigated and confirmed in experimental *in vivo* systems.

Since IL-12/15/18 pre-activated NK cells are sufficient in antibody-mediated cytotoxicity despite partial CD16 shedding (35, 47, 63), a scenario of NK cell activation through both CD16 and IL-12 may synergistically improve NK cell activity like IFN-γ production (64). It has been inferred from mouse and human studies that tumor-reactive therapeutic antibodies may promote uptake and presentation of tumor antigens by dendritic cells, resulting in the formation of antigen-specific T cell memory (65–67). In contrast, the physiological existence and role of CD16-induced memory-like NK cells *in vivo* is currently unknown. However, it would require the presence of ADCC-sufficient therapeutic antibodies or killer engagers applied in cancer patients (68). In this context, CD16-induced memory-like NK cell functionality might be preferentially induced in patients carrying the high affinity CD16-158V/V genotype, which confers better clinical efficacy in cancer patients treated with IgG-type therapeutic antibodies (69, 70). Moreover, CD16-induced memory-like NK cells might support T/B cell responses during primary infection or reinfection, when endogenous IgG antibodies are produced e.g., against HCMV (71–73). While (HCMV-specific) antibodies have been shown to promote the expansion of pre-existing HCMV-associated NKG2C^+^ “adaptive” NK cells (74–76), antibodies probably do not mediate in the initial generation of NKG2C^+^ “adaptive” NK cells in HCMV-seronegative individuals (76). In contrast to the NKG2C^+^ “adaptive” NK cells (75), CD16-induced memory-like NK cells maintained expression of FcεR1γ and NK cell activating receptors and exerted enhanced IFN-γ in response to IL-12 and enhanced antibody-independent cytotoxicity (47). It is unknown whether HCMV-specific antibodies can induce CD16-induced memory-like NK cell functionality. Finally, it is unknown whether other ITAM-coupled activating receptors or chimeric antigen receptors in human NK cells have the potential to induce memory-like functionality similar to FcγRIII/CD16. In this regard, engagement of the murine ITAM-coupled activating receptor Ly49H has been shown to mediate the expansion of virus/m157-specific memory NK cells in mice with long-lasting enhanced responsiveness to secondary stimulation (9).

## Epigenetic regulation

NK cells intrinsically “remember” a previous exposure to cytokines, since IL-12/15/18 pre-activated NK cells pass their enhanced IFN-γ producing capacity to daughter cells (39, 42). Epigenetic imprinting, e.g., demethylated CNS1 region of the *IFNG* gene, was detected in HCMV-associated NKG2C^+^ “adaptive” NK cells (75, 77) and IL-12/15/18 pre-activated NK cells (42) (Figure 1), which was shown to lead to a remarkable stability of the IFN-γ-producing phenotype even after adoptive transfer. Both IL-12 and IL-18 are essential for the pronounced demethylation of the CNS1 region (42, 77), while IL-15 might serve as a survival factor. Besides the *IFNG* gene, CpG demethylation of the *PRDM1/BLIMP1* and *ZBTB32/TZFP* genes or hypermethlyation of *FCER1G* (Fc fragment of IgE receptor Ig) were also detected in NKG2C^+^ “adaptive” NK cells (75, 77). Recently, stable epigenetic changes were also found in MCMV-specific memory NK cells, some of which are shared by memory CD8 T cells (78). Particularly, IL-12 during MCMV infection induces epigenetic remodeling of the *IRF8* gene, an important regulator for the proliferation of MCMV-specific NK cells (79). This finding sheds light on NK cell deficiencies in individuals with *IRF8* mutations associated with severe viral infections (80). However, it is not clear how IRF8 intrinsically/extrinsically affects NK cell function (that is impaired in IRF8^−/−^ patients), or the formation and maintenance of the long-lived MCMV-specific NK cell memory compartment including protection against re-infection.

## Metabolic regulation

Metabolic regulation is pivotal for the development, maintenance and recall responses of memory T cells (81). Similar to T cells, activation of NK cells by cytokines such as IL-2, IL-15, IL-12, and IL-18 or activating receptors lead to elevated oxidative phosphorylation (OXPHOS) and elevated glycolysis (82, 83). The *in vitro* IFN-γ production from IL-12/18 stimulated NK cells was not affected by inhibition of glycolysis and mitochondrial OXPHOS under certain *in vitro* activating condition (83). In fact, increased rates of glycolysis are required for NK cell mediated control of MCMV infection (84). Hence, it is of interest whether increases in glycolysis regulate the generation and function of IL-12/15/18-induced memory-like NK cells including their recall responsiveness.

The Srebp-controlled increased citrate-malate shuttle is required for elevated glycolysis and oxidative phosphorylation in cytokine-stimulated NK cells (85). Anti-tumor activity of adoptively transferred IL-12/15/18 pre-activated NK cells was lost when Srebp inhibitors were present during NK cell activation, suggesting the importance of the citrate-malate shuttle in cytokine-induced memory-like function of NK cells. The citrate-malate shuttle exports acetyl-CoA into the cytosol via citrate. Of note, it was shown in a recent study that histone acetylation was controlled by changing levels of nuclear acetyl-CoA (86). This finding links the metabolic regulation to the epigenetic modification, which might be crucial for the persistent competence of enhanced gene transcription upon restimulation. Further investigation of the impact of acetyl-CoA on histone modification in NK cells would help to reveal the possible association of the NK cell memory-like phenotype with cytokine-induced metabolic changes (Figure 1).

Recently, it has been shown that the fundamental metabolic regulator cMyc controls metabolic and functional activation of NK cells upon cytokine stimulation (87). Whether cMyc is involved in regulating NK cell memory similar as in memory CD8 T cells awaits further investigation (88).

## Physiological relevance

The physiological relevance and existence of human memory-like NK cells *in vivo* remains to be resolved. In mice, long-lived NK cells were generated during respiratory syncytial virus infection that undergo homeostatic proliferation or virus-induced proliferation in the bone marrow but not at the primary sites of infection like respiratory tissues (89). In mice, IL-12, IL-18 are essential co-stimulatory factors for the generation of murine CMV-specific Ly49H^+^ memory NK cells (11). Furthermore, IL-12 and IL-18 are important for the expansion of HCMV-associated NKG2C^+^ “adaptive” NK cells *in vitro* (12, 73). Hence, a coordinated availability of IL-12, IL-15, and IL-18, derived from dendritic cells and myeloid cells during viral infections such as CMV or influenza (90), might support generation of cytokine-induced memory-like NK cells *in vivo*. Of note, a study of tracking the fate of NK cells upon MCMV infection showed the induction of both antigen-specific memory Ly49H^+^ NK cells and cytokine-activated antigen-unspecific long-lived Ly49H^−^ NK cells, and the differentiation of both subsets critically relied on IL-12. This study highlighted the *in vivo* relevance of cytokine-induced memory-like features in NK cells. These cytokine-activated persistent Ly49H^−^ NK cells were less responsive to restimulation by activating receptors *in vitro* or tumor cells *in vivo* but survived longer in an MCMV-free environment (91).

Interestingly, durable enhanced IFN-γ responses by NK cells (and NKT cells) have been reported in humans up to 1 year after Bacillus Calmette-Guérin (BCG) revaccination in response to BCG restimulation, at a time point when BCG-specific IL-2 producing CD4 T cells were reduced (92). The enhanced NK cell IFN-γ response involved IL-12 and IL-18, probably derived from myeloid peripheral blood cells. The contribution of IL-2 was low, suggesting that IL-2 producing memory T cells were dispensable in BCG infection (92), unlike in certain viral infections (19, 20). IL-21 can potentiate the expansion and anti-tumor activity of IL-2 stimulated NK cells (93). In mice, BCG vaccination was suggested to generate long-lived NK cells, which possessed high proliferative capacity and anti-bacterial activity when restimulated with the mycobacteria tuberculosis antigen complex (94). Notably, this observation required the presence of T/B cells or IL-21 during BCG vaccination. Overall, it remains to be determined whether BCG-reactive NK cells have indeed intrinsic memory-like functionality and/or require the contribution of myeloid cells, which are known to undergo epigenetic modifications and immune training (akin to innate immune memory) in response to BCG vaccination (95, 96). Mechanistically, it will be of relevance to determine whether BCG-induced memory NK cells share regulatory mechanisms such as epigenetic imprinting with cytokine-induced memory-like NK cells or MCMV-specific memory NK cells (8, 78).

## Clinical potential

It has been demonstrated in different mouse and rat tumor models that IL-12/15/18 pre-activated NK cells can confer favorable therapeutic effects after adoptive cell transfer, such as enhanced IFN-γ production, cytotoxicity, and long-lived capacity for antigen-unspecific immunological memory (40–42, 44) (Table 1). Importantly, IL-12/15/18 pre-activated NK cells have been suggested to alleviate severe acute graft-vs.-host disease (GvHD). In a mouse model of a fully mismatched hematopoietic cell transplant, co-transfer of autologous IL-12/15/18 pre-activated NK cells limited the severity of acute GvHD by presumably restricting the proliferation of adoptively-transferred allogeneic T cells while the graft-vs.-leukemia (GvL) effect of the T cells was retained (Table 1) (97). Still, the role of IL-12/15/18 pre-activated NK cells on delayed or failed stem cell engraftment and development of chronic GvHD remains incompletely understood (97). Consistent with the previous study it has been recently reported that adoptive transfer of murine NK cells pre-activated with IL-12/18+/− IL-15, which had been expanded with IL-2 before, suppressed severe acute GvHD (Table 1) (98). However, IL-12/15/18 pre-activated NK cells, in contrast to IL-12/18 pre-activated NK cells, did not have a protective effect against mild acute GvHD. The absence of a potent direct anti-tumor activity of IL-12/15/18 pre-activated NK cells in these mouse studies might be due to the lack of host CD4 T cells and insufficient IL-2 that may be instrumental in maintaining durable anti-tumor responses (40). Hence, with respect to the variegated NK cell pre-activation protocols, it needs to be refined how the different cytokine pre-activation protocols, dosing regiments and *in vivo* cytokine supplementation maximize GvL effects and minimize acute and chronic GvHD.

**Table 1:** *In vivo* therapeutic application of memory-like NK cells.

**System**	**Tumor entity**	**Type of NK cells**	**Therapeutic strategy**	**Therapeutic effect**	**References**
Rodent	Mouse T-cell lymphoma RMA-S	IL-12/15/18 NK	Day 7: TBI and 1 × 10^6^ NK cells	+++	(40)
	Mouse B16 lung metastasis	IL-12/15/18 NK	Day 7: TBI and 1 × 10^6^ NK cells	+++	(40)
	Mouse B16 Melanoma	IL-12/15/18 NK	Day 3, 7, 10: 1.2–2 × 10^6^ NK cells	++	(85)
	Mouse allo-HSCT and GVL (B-cell lymphoma A20)	IL-12/15/18 NK	Day 0: 1 × 10^6^ NK cells	+++	(97)
	Mouse allo-HSCT and GVL (B-cell lymphoma A20)	IL-12/18 BM-NK IL-12/15/18 BM-NK	Day 0: 5 × 10^6^ NK cells; or day 0, 7, 14: 5 × 10^6^ NK cells	++/+++	(98)
	Rat T-ALL/Roser Leukemia	IL-12/15/18 NK	TBI and day 3, 6, 9: 4–6 × 10^6^ NK cells	+++	(41)
Xenograft	Leukemia K562	IL-12/15/18 NK	Day 4: 5 × 10^6^ NK cells and IL-2 i.p.	+++	(44)
	Melanoma SK-Mel-28	IL-12/15/18 NK	Day 0: 1–3 × 10^6^ NK cells and IL-2 i.p.	+++	(42)
Human	AML phase I; 9 patients	Allogeneic IL-12/15/18 NK	FC and 0.5 ×, 1 × or 10 × 10^6^ NK cells plus IL-2	5 clinical responses including 4 complete remission	(44)

*IL-12/15/18 NK, IL-12/15/18 pre-activated NK cells; TBI, total body irradiation; GVHD, Graft-vs.-host disease; allo-HSCT, allogeneic hematopoietic stem cell transplantation; BM-NK, bone-marrow derived NK; FC, fludarabine/cyclophosphamide*.

In patients with AML it has been shown that alloreactive NK cells generated from haploidentical hematopoietic stem cell grafts reduced leukemia recurrence and lowered the risk for GvHD while contributing to GvL effects (99). Recently, Romee et al. has pioneered the adoptive transfer of haploidentical IL-12/15/18 pre-activated memory-like NK cells in a phase I clinical study in nine heavily pre-treated relapsed/refractory AML patients (Table 1). In this trial, IL-12/15/18 pre-activated NK cells displayed an enhanced proliferative state, leading to increased frequencies in the recipients after IL-2 supplementation *in vivo* (44). Importantly, 7 days after adoptive transfer, IL-12/15/18 pre-activated NK cells exerted potent anti-tumor activity *ex vivo* after restimulation, correlating with improved survival in the absence of GvHD in a subset of AML patients. Thus, adoptive transfer of human IL-12/15/18 pre-activated memory-like NK cells into AML patients with active disease is considered safe and feasible, resulting in donor NK cell expansion, GvL activity and favorable clinical responses. Thus, this study initiated a promising translational immunotherapy approach for durable NK cell anti-cancer responses not only for AML but also for other NK cell-sensitive tumors. Overall, the use of donor NK cells for adoptive transfer may be more favorable than autologous NK cells from cancer patients, which are often functionally impaired or less responsive to cytokine activation (25, 100, 101).

## Conclusion

Pre-activation of NK cells by the cytokines IL-12/18 plus IL-15 or by engagement of FcγRIII/CD16 via therapeutic antibodies can induce similar memory-like functionalities: an enhanced proliferative capacity toward IL-2 due to CD25 up-regulation as well as a strengthened responsiveness to restimulation by tumor cells. Importantly, both memory-like functionalities are antigen-unspecific and imply “remembering” a previous state of heightened activation induced by cytokine exposure or stimulation via activating NK cell receptors. These memory-like functionalities have unveiled a previously unappreciated potential for NK cell-based cancer immunotherapy. Several aspects may improve the translation of the recent findings into clinical application. First, clear criteria for NK cell-sensitive tumors are pivotal, which requires proper genetic and protein markers. Second, optimal NK cell activation protocols as well as pre-conditioning regimens of patients need to be established to improve engraftment, expansion, persistence and durable anti-cancer activity after adoptive transfer. Third, epigenetic and metabolic parameters should be monitored and manipulated during cancer therapy to sustain NK cell reactivity in the tumor microenvironment. In future clinical studies, it remains to be determined whether clinical responses by memory-like NK cells may be augmented by the combination with therapeutic tumor-reactive antibodies or checkpoint immunotherapy.

## Author contributions

All authors listed have made a substantial, direct and intellectual contribution to the work, and approved it for publication.

### Conflict of interest statement

JP and AC received a commercial research grant from Affirmed. AC is a consultant/advisory board member for SAB Dragonfly Therapeutics. The remaining author declares that the research was conducted in the absence of any commercial or financial relationships that could be construed as a potential conflict of interest. The handling editor declared a past co-authorship with AC.
